# Efficacy of two intensive and spaced protocols of theta burst transcranial magnetic stimulation in treatment-resistant depression: a double-blind randomized trial

**DOI:** 10.1192/j.eurpsy.2023.1270

**Published:** 2023-07-19

**Authors:** Y. Cañada, P. Navalón, P. Benavent, A. Sabater, J. Ribes, L. Livianos, P. Sierra

**Affiliations:** 1 Mental Health Group, La Fe Health Research Institute; 2 Psychiatry, La Fe University and Polytechnic Hospital, Valencia, Spain

## Abstract

**Introduction:**

Depression is the disease with the greatest burden of disability. Despite pharmacological options, up to 30% of depressions are considered resistant to treatment (RTD). Theta burst transcranial magnetic stimulation (TBS) on the dorsolateral prefrontal cortex allows the application of shorter protocols and with longer-lasting effects than conventional TMS. Its implementation in the Public Health System requires the design of efficient, cost-effective and accessible protocols for patients.

**Objectives:**

The objective of this project is to assess the efficacy and safety of two intensive and spaced protocols (unilateral and bilateral) of 1800 pulses TBS compared to sham stimulation in outpatients with unipolar and bipolar TRD in a public hospital.

**Methods:**

This project is the 1st double-blind placebo-controlled RCT with TBS in Spain. It is now in the recruitment phase. Patients receive a total of 22 sessions of 1800 pulses in 6 weeks: 5 days/week the 1st and 2nd weeks (10 sessions) and 3 sessions/week the following weeks (12 sessions). Patients are randomized into three groups: i) bilateral, ii) left unilateral, and iii) sham. The main variable is the change in the HDRS-17 score at the end of treatment compared to baseline. The results were analyzed with a general linear model of HDRS, using time as the intrasubject factor and randomization group as the intersubject factor, using resistance to treatment (Maudsley Score) and diagnosis (bipolar, unipolar) as covariates.

**Results:**

Preliminary results from 13 participants (nbil=4, nuni=4, nsham=5) reveal a significant effect of (group x time) on HDRS change (p= 0.020) with no influence of Maudsley Score or diagnosis. The bilateral group presented a greater decrease in the HDRS with a mean difference of 4,38 points [CI95% 0,17-8,58), (p=0.043)] with respect to the unilateral group and a difference of 8.23 [CI95% 4,24-12,21)(p =0.001)] compared to the sham group.

**Table 1:** Sample description. Data shown are means and standar deviations. In bold significant diferences p<0,05.

	Bilateral TBS (n=4)	Unilateral TBS (n=4)	Sham TBS (n=5)
Age [M (SD)]	55,50 (5,80)	48,50 (16,86)	56,40 (5,86)
Male: Female [n]	0:4	2:2	3:2
Depression:Bipolar disorder [n]	3:1	3:1	4:1
Length of depresive episode (months) [M (SD)]	12,00 (4,08)	16,50 (6,61)	15,00 (5,83)
Current number of antidepressants [M (SD)]	2,00 (0,82)	1,75 (0,50)	2,60 (0,55)
Maudsley score [M (SD)]	7,00 (1,82)	8,75 (1,5)	7,90 (1,30)
Basal HDRS basal [M (SD)]	16,00 (1,82)	19,75 (4,03)	20,40 (3,21)
Final HDRS [M (SD)]	**5,75** **(3,30)**	**10,75** **(3,10)**	**17,80** **(2,49)**
Response /Remission[n (%)]	3:**3** (75%)	2:**0(**50:0%)	0:**0 (**0%)

**Image:**

**Figure:**
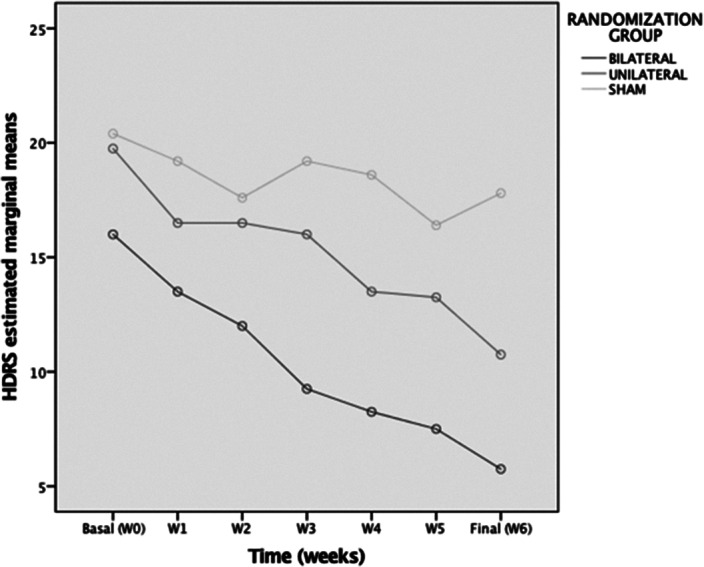

**Conclusions:**

The results demonstrate the preliminary efficacy of intensive TBS protocols relative to sham.

**Disclosure of Interest:**

None Declared

